# Perioperative morbidity and midterm outcomes following antireflux surgery in overweight and obese populations

**DOI:** 10.1007/s00464-026-13263-w

**Published:** 2026-08-06

**Authors:** Andrés R. Latorre-Rodríguez, Arianna Vittori, Hideyuki Takeuchi, Sumeet K. Mittal

**Affiliations:** 1 Norton Thoracic Institute, St. Joseph’s Hospital and Medical Center, 500 W Thomas Road, Phoenix, AZ 85013 USA; 2 Universidad del Rosario, Escuela de Medicina y Ciencias de La Salud, Grupo de Investigación Clínica, Bogotá D.C., Colombia; 3 Department of Surgery, Oncology and Gastroenterology, School of Medicine, University of Padua, Padua, Italy; 4 Department of Surgery, The Jikei University School of Medicine, Tokyo, Japan; 5 Creighton University School of Medicine, Phoenix, AZ USA

**Keywords:** Antireflux surgery, Gastroesophageal reflux disease, Obesity, Overweight, Patient-reported outcomes

## Abstract

**Background:**

As the worldwide prevalence of obesity increases, more patients with obesity are referred for antireflux surgery (ARS). Although bariatric surgery is typically recommended, at times straightforward ARS is offered. We aimed to compare surgical and patient-reported outcomes among overweight and obese patients undergoing minimally invasive ARS.

**Methods:**

This retrospective cohort study included consecutive patients with BMI ≥ 25 kg/m^2^ who underwent primary elective ARS by an experienced foregut surgeon (September 2016–June 2025). Patients were stratified by BMI: overweight 25–30, class I obesity 30–35, class II obesity 35–40, and class III obesity ≥ 40 kg/m^2^. Exclusions included prior foregut procedures or non-fundoplication ARS (e.g., magnetic sphincter augmentation). Outcomes of interest were perioperative morbidity, hernia recurrence, midterm quality-of-life scores, and patient satisfaction.

**Results:**

A total of 338 patients (71.6% female; median age: 65.1 [54.6–73.1] years) were stratified into the BMI groups: overweight (*n* = 172, 50.9%), class I (*n* = 125, 36.9%), class II (*n* = 30, 8.9%), and class III (*n* = 11, 3.3%). Intraoperative complication rates (3.5, 4.8, 3.3, and 27.3%; *p* = 0.006) and ICU admission rates (1.7, 4, 3.3, and 18.2%; *p* < 0.026) were significantly higher, and early postoperative complications were slightly higher (7.6, 8.8, 6.7, and 27.3%; *p* = 0.153) in the class III obesity group. Follow-up ≥ 12 months was available for 95/172 (55.2%), 64/125 (51.2%), 15/30 (50%), and 3/11 (27.3%) patients per group with similar median durations (24–36 months). Objective hiatal hernia recurrence (> 2 cm) was observed in 1 overweight and 2 class-I patients, but no class-II/III patients. Improved quality-of-life (GERD-HRQL) scores and high patient satisfaction, especially in the 25–40 kg/m^2^ BMI groups, were reported.

**Conclusion:**

Minimally invasive ARS provides a favorable safety profile along with gastroesophageal reflux control and improved quality of life for overweight and non-morbidly obese patients. Particularly, straightforward ARS for patients with class II obesity may be considered when limitations on bariatric surgery exist.

**Graphical abstract:**

**Figure:**
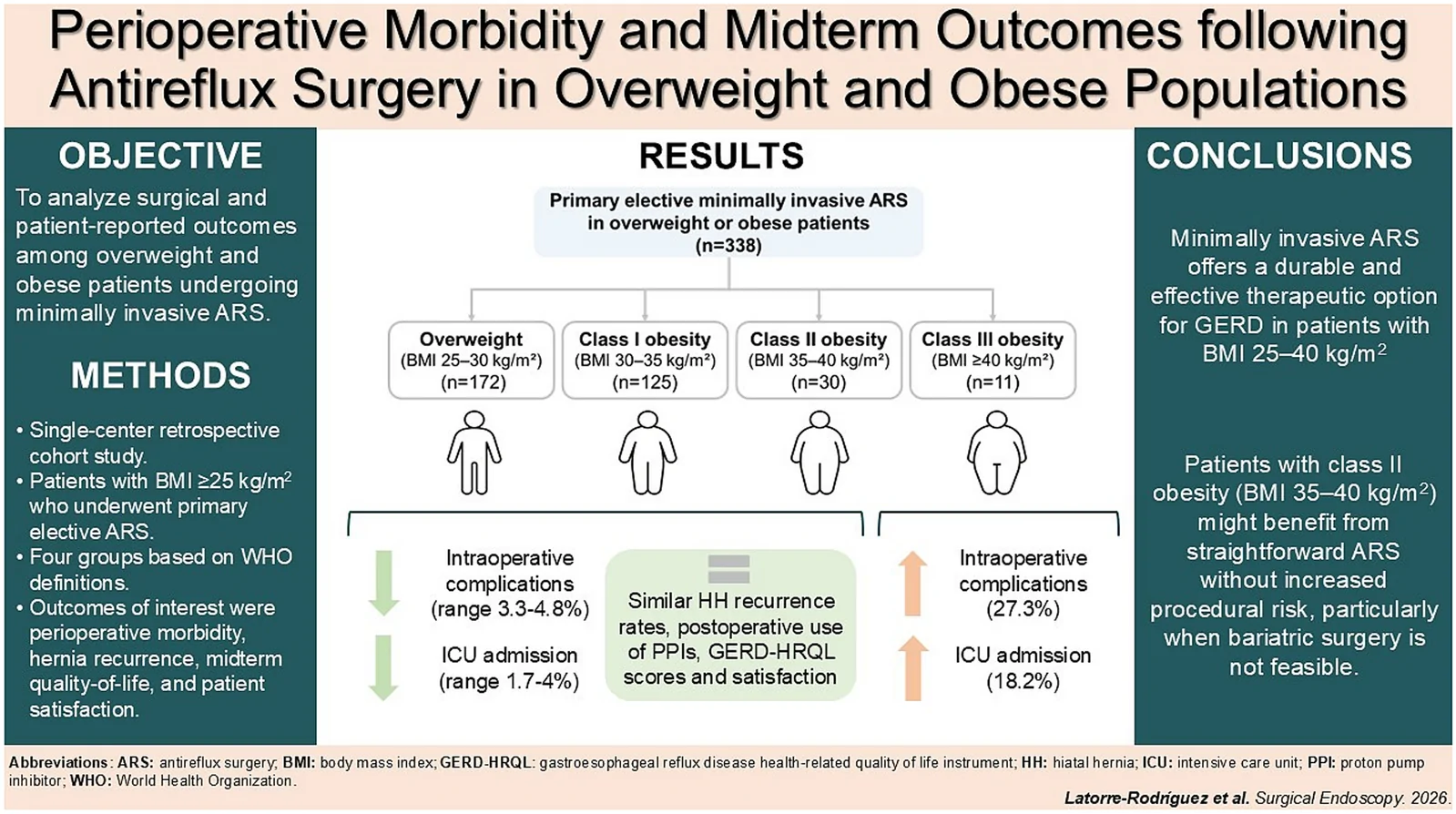

**Supplementary Information:**

The online version contains supplementary material available at https://doi.org/10.1007/s00464-026-13263-w.

The rapidly increasing prevalence of obesity and its related metabolic conditions constitutes a major global public health challenge [1]. At present, obesity affects 40.3% of adults in the United States with projected growth to ~ 50.5% by 2060 [2, 3]. This demographic shift is accompanied by an increasing incidence of other chronic conditions such as gastroesophageal reflux disease (GERD), and compelling evidence points to obesity as a key contributor to GERD development [4]. The interplay between obesity and GERD is multifactorial, including mechanisms such as elevated intra-abdominal pressure, altered transabdominal pressure gradients, and higher rates of transient lower esophageal sphincter relaxations, all of which facilitate reflux [5–7]. Furthermore, increased abdominal visceral adipose tissue may contribute to systemic inflammation and local disruption of the esophageal mucosal barrier [8].

Consequently, obesity introduces unique complexities in terms of safety and effectiveness for GERD management. Although lifestyle modifications and acid suppression therapy remain the first-line therapeutic approaches for symptom control among overweight and obese populations [9], these strategies are often considered unsustainable in the long term as they do not fully address the underlying mechanisms; thus, consideration of definitive surgical treatment is recommended in the 2022 American College of Gastroenterology Clinical Guidelines as well as in the 2023 multi-society consensus (SAGES, EAES, SSAT, STS, ASGE, ASMBS) [9, 10].

On one hand, there is overall agreement that Roux-en-Y gastric bypass (RYGB) provides a significant metabolic advantage and durable weight control, thereby positioning it as a preferred, albeit more complex, alternative for severely and morbidly obese patients (body mass index [BMI] ≥ 35 kg/m^2^) [9, 11]; however, its perceived perioperative risks, resource utilization (i.e., costs), requirement for strict adherence to bariatric programs, and often limited insurance coverage frequently restrict patient eligibility and candidacy [12, 13]. On the other hand, minimally invasive antireflux surgery (ARS), specifically laparoscopic partial fundoplication, is the established gold standard for GERD treatment among non-obese individuals due to its safety profile and excellent long-term efficacy [14]; however, its outcomes in overweight and obese cohorts have been questioned since the early laparoscopic era [15, 16]. Specific concerns include a hypothesized increased risk of fundoplication failure (e.g., wrap disruption, re-herniation) in the absence of sustained weight loss as well as potentially higher morbidity in this population [16, 17]. Thus, we aimed to describe and compare the surgical outcomes and patient-reported outcomes (quality-of-life and satisfaction) across overweight and obese patients (i.e., BMI ≥ 25 kg/m^2^) who underwent elective minimally invasive laparoscopic fundoplication.

## Methods

### Study design, data source, and setting

We conducted a retrospective observational cohort study using a prospectively maintained database of all patients who underwent antireflux procedures performed by the senior author at a tertiary care facility in the Phoenix, Arizona, U.S.A. Data on demographics, surgical details, and outcomes of procedures completed between September 2016 and June 2025 were retrieved from this dataset. This project was approved by the Institutional Review Board of St. Joseph’s Hospital and Medical Center under the Norton Thoracic Institute Foregut Umbrella Protocol (PHXU-21-500-136-73-18). Patient consent was waived given the retrospective nature of the study. All study activities adhered to the 2024 Revision of the Declaration of Helsinki, and results were reported following the Strengthening the Reporting of Observational Studies in Epidemiology (STROBE) statement and checklist (Supplementary Material–S1).

### Study population and study groups

All adult patients (≥ 18 years) with a BMI ≥ 25 kg/m^2^ who underwent primary elective minimally invasive ARS (laparoscopic or robotic) between September 2016 and June 2025 were eligible for inclusion. Exclusion criteria included: (i) a history of previous major foregut procedures, (ii) revisional surgery, (iii) patients who underwent antireflux procedures other than fundoplication (e.g., Roux-en-Y gastric bypass, magnetic sphincter augmentation, transoral incisionless fundoplication, etc.), (iv) those who received additional concomitant procedures (e.g., pyloroplasty, cholecystectomy, abdominal wall hernia repair, J or G tube, etc.), and (v) surgery performed on lung transplant candidates or recipients.

To assess the impact of BMI on perioperative morbidity and midterm outcomes, patients were stratified into four groups based on specific cut-offs, according to the World Health Organization definitions: overweight (25–30 kg/m^2^); class I obesity (moderately obese, 30–35 kg/m^2^); class II obesity (severely obese, 35–40 kg/m^2^); and class III obesity (morbidly obese, ≥ 40 kg/m^2^).

### Clinical care and surgical details

The standard preoperative workup and clinical care offered to patients undergoing primary elective ARS at our institution have been previously reported [18]. Overall, all patients underwent preoperative esophagogastroduodenoscopy (EGD), barium esophagram, and functional testing if indicated (e.g., esophageal high-resolution manometry, pH monitoring, gastric emptying studies, endoscopic functional luminal impedance planimetry [EndoFLIP]). Of note, patients with GERD and obesity class II/III are generally directed toward metabolic bariatric surgery (i.e., RYGB) regardless of the presence or severity of obesity-related comorbidities; however, at times we offer straightforward ARS due to significant limitations in insurance coverage, patient preferences, inability of the patient to fulfill the requirements of a comprehensive bariatric program, or other administrative issues.

The antireflux procedure and its approach is tailored based on the preoperative workup findings and patient preferences; nevertheless, whether using conventional laparoscopy or a robotic-assisted platform (DaVinci Xi, Intuitive Surgical), the surgical technique follows a reproducible approach including (i) hernia sac excision and reduction, (ii) mediastinal mobilization, (iii) correction of crural defect using non-absorbable sutures, occasionally supplemented by crural reinforcement with a biosynthetic mesh (particularly in cases of a large hiatal hernia [HH] or intrathoracic stomach); and (iv) creation of a partial fundoplication. In most cases, a 300° symmetrical anterior–posterior partial (Lind-like) fundoplication incorporating Angle of His (AOH) accentuation is created. In other cases, especially when some degree of preoperative dysphagia is reported, a 180° anterior Dor fundoplication or AOH accentuation alone may be preferred.

Postoperatively, patients are observed overnight, and in the absence of postoperative complications, discharge occurs on postoperative day 1. The initial postoperative visit is scheduled at 1–2 weeks, followed by a second visit at 5–7 weeks. Subsequent appointments occur at 1, 2, and 3 years after surgery, and then biennially. Scheduled follow-up visits are conducted either in-person or remotely and include anamnesis, a barium swallow study, and the administration of validated survey instruments, such as the modified Gastroesophageal Reflux Disease Health-Related Quality of Life (GERD-HRQL) questionnaire. In cases where patients present with symptom recurrence or any other concerning issue, unscheduled visits are accommodated and further diagnostic tests are ordered as indicated.

### Outcomes and definitions

Outcomes of interest in relation to *perioperative morbidity and procedure safety* included intraoperative complications (e.g., organ laceration/perforation, bleeding, etc.), hospital length of stay (LOS), rates of early postoperative complications (classified by the Clavien–Dindo system and occurring during the index hospitalization), and intensive care unit (ICU) admissions directly attributable to the procedure.

Moreover, outcomes related to *midterm surgical effectiveness* included objective hiatal hernia recurrence (defined as a re-herniation > 2 cm noted on barium esophagram or EGD) at any time after surgery, patient-reported quality of life (assessed via the modified GERD-HRQL instrument), and overall patient satisfaction. Patient satisfaction was assessed using an elective follow-up question which includes a visual analog scale from 0 (complete dissatisfaction) to 10 (maximum possible satisfaction), with a score of ≥ 8 indicating acceptable satisfaction. For both quality-of-life and patient satisfaction metrics, data were retrieved from the last available follow-up occurring at least 12 months after surgery.

### Statistical analysis

All consecutive patients meeting the study inclusion criteria were included. Continuous variables are presented as median (interquartile range [IQR]) and categorical variables as count (proportion). Non-normal distribution of continuous data was confirmed using the Shapiro–Wilk test and visual inspection of Q–Q plots; thus, group differences in continuous variables were subsequently analyzed using the Kruskal–Wallis test. For categorical variables, group differences were analyzed using the Pearson’s chi-squared test. To control the risk of Type I error derived from multiple comparisons, post hoc Dunn’s test or Holm–Bonferroni correction was applied as appropriate. Missing data were handled using available case analysis (i.e., adjustment of denominators). A two-sided alpha level of 0.05 was set to denote statistical significance. All analyses were completed using R version 4.4.1 (Foundation for Statistical Computing, Vienna, Austria).

## Results

### Baseline characteristics

**Fig. 1:**
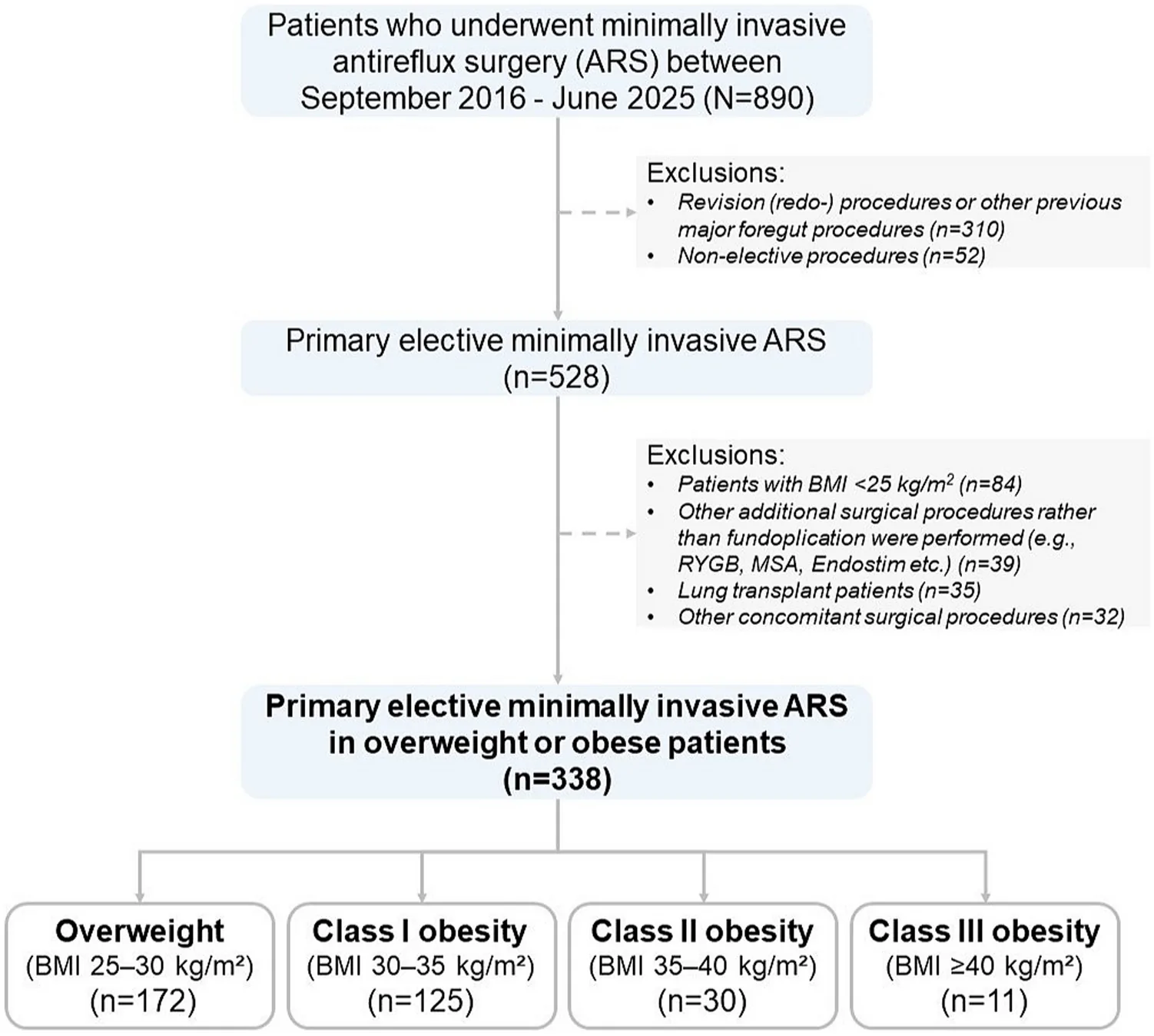
Study flow diagram

A total of 528 patients underwent primary elective minimally invasive ARS during the study period and were assessed for eligibility. Patients with BMI < 25 kg/m^2^ (*n* = 84), those who received non-fundoplication antireflux procedures (*n* = 39) or concomitant procedures (*n* = 32), and those with advanced respiratory disease (i.e., lung transplant patients; *n* = 35) were excluded from analysis. The observed prevalence of overweight (25–30 kg/m^2^) and obesity (> 30 kg/m^2^) among patients undergoing elective primary ARS was 32.6% (95% CI: 28.6–36.8%) and 31.4% (95% CI: 27.5–35.6%), respectively. Therefore, the analyzed cohort consisted of 338 patients. Figure 1 presents the study flow diagram.

Overall, most patients were female (*n* = 242 [71.6%]), the median age was 65.1 (54.6–73.1) years, and the median BMI was 29.9 (27.6–32.7) kg/m^2^. Of note, 270 (80.6%) cases were completed via conventional laparoscopy, 64 (19.1%) via robotic-assisted surgery, and 1 (0.3%) patient required conversion to open surgery due to extensive adhesions. Surgical techniques included 270 (79.9%) symmetrical anterior–posterior partial (Lind-like) fundoplications, 41 (12.1%) anterior Dor fundoplications, and 27 (8%) AOH accentuation procedures alone. Moreover, crural reinforcement was completed in 81 (24%) cases using biological resorbable mesh.

Baseline demographic and surgical characteristics, stratified by BMI, are summarized in Table 1. Notably, there was a significantly greater proportion of females in the class III obesity group than in the overweight group (90.9 vs. 64.5%). Similarly, patient age presented a weak inverse relationship with BMI (*r*_*s*_ = –0.171, *p* = 0.001), with patients in the class III obesity group being significantly younger than those in the overweight group (58.5 vs. 68 years). Conversely, although typical GERD/volume reflux was the most common indication for surgery among patients with a BMI between 25 and 40 kg/m^2^, most patients with class III obesity primarily presented with obstructive symptoms (54.5%); however, this difference did not achieve overall statistical significance across all groups. Furthermore, despite broadly similar preoperative rates of acid suppression therapy across the groups, a slightly higher use of proton pump inhibitors (PPIs) was observed among the class I obesity group compared to the overweight group (89.8 vs. 72.4%). All other baseline characteristics, including the preoperative total GERD-HRQL score, surgical approaches, specific surgical techniques employed, and the dimensions of the diaphragmatic defect, were similar across the groups.

**Table 1:** Baseline characteristics stratified by body mass index categories

	Body mass index—obesity class				
	Overweight 25–30 kg/m^2^ (*n* = 172)	Class I 30–35 kg/m^2^ (*n* = 125)	Class II 35–40 kg/m^2^ (*n* = 30)	Class III ≥ 40 kg/m^2^ (*n* = 11)	*p* value
Demographics					
Sex, female	111 (64.5)	96 (76.8)	25 (83.3)	10 (90.9)*	**0.019**
Age, years	68 [56.4–74]	63.5 [54–71.6]	62.1 [50.4–70.4]	58.5 [47.4–64]*	**0.028**
BMI, kg/m^2^	27.7 [26.2–28.8]	32.2 [31.2–33.3]*	36.3 [35.6–38.2]*	41.1 [40.4–42.7]*	** < .001**
Primary surgical indication					
Typical GERD/volume reflux	104 (60.5)	83 (66.4)	17 (56.7)	2 (18.2)	0.148
Obstructive symptoms	46 (26.7)	25 (20)	7 (23.3)	6 (54.5)	
Chronic bleeding/anemia	14 (8.1)	14 (11.2)	4 (13.3)	2 (18.2)	
Atypical GERD symptoms	8 (4.7)	3 (2.4)	2 (6.7)	1 (9.1)	
Quality of life and medical therapy					
Completed GERD-HRQL	116 (67.4)	79 (63.2)	19 (63.3)	8 (72.7)	0.827
Total GERD-HRQL score†	26.5 [12.8–45.3]	32 [14.5–46]	30 [20–52]	42 [23.8–48.5]	0.399
Any acid suppression therapy†	97/116 (83.6)	71/79 (89.8)	16/19 (84.2)	5/8 (62.5)	0.182
H2-receptors antagonist	19/116 (16.4)	6/79 (7.6)	1/19 (5.3)	2/8 (25)	0.148
Proton pump inhibitors	84/116 (72.4)	71/79 (89.8)*	16/19 (84.2)	4/8 (50)	**0.005**
Surgical approach					
Laparoscopic	136 (80)	100 (80.6)	24 (80)	10 (90.9)	0.864
Robotic	34 (20)	23 (18.5)	6 (20)	1 (9.1)	
Conversion to open	0 (0)	1 (0.8)	0 (0)	0 (0)	
Surgical procedures					
Mesh reinforcement	41 (23.8)	27 (21.6)	9 (30)	4 (36.4)	0.591
300° partial Lind-like fundoplication	143 (83.1)	98 (78.4)	22 (73.3)	7 (63.6)	0.551
180° partial Dor fundoplication	16 (9.3)	17 (13.6)	5 (16.7)	3 (27.3)	
Angle of His accentuation alone	13 (7.6)	10 (8)	3 (10)	1 (9.1)	
Intraoperative findings					
Stomach in the thorax, %	33 [11.3–75]	30 [10–50]	36.5 [17.5–68.3]	50 [20–90]	0.424
Crus antero-posterior dimension, cm	4 [4, 5]	4 [4, 5]	4 [4, 5]	4.8 [4.5–5]	0.277
Crus transverse dimension, cm	3 [2–4]	3 [2–3.5]	3 [3, 4]	2.8 [2.5–3.8]	0.216
Abdominal esophageal length, cm	2 [1.5–2.4]	2 [1.5–2]	2 [1.5–2]	2 [1.5–2]	0.778

Bold *p* values indicate a *p* < 0.05

*BMI* body mass index, *GERD-HRQL* Gastroesophageal Reflux Disease Health-Related Quality of Life

^*^Indicates an adjusted *p* < 0.05 (via Holm–Bonferroni correction or Dunn’s test) when compared to patients with a BMI between 25 and 30 kg/m^2^

^†^Reported frequencies are adjusted by the denominators of available complete GERD-HRQL data

### Procedure safety and perioperative morbidity

Table 2 presents the detailed aspects of procedure safety and perioperative morbidity. The median operative time (ranging between 87.5 and 110 min) was overall similar across the groups, although it was slightly higher in the class III obesity group. Moreover, patients with class III obesity exhibited greater median estimated blood loss compared to overweight patients (37.5 vs. 20 mL); however, only 2 patients (1 each in the class I and class III groups) experienced significant bleeding (≥ 250 mL). Intraoperative complication rates (overweight: 3.5%; class I: 4.8%; class II: 3.3%; and class III: 27.3%; *p* = 0.006) and ICU admission rates (overweight: 1.7%; class I: 4%; class II: 3.3%; and class III: 18.2%; *p* = 0.026) were greater in the class III obesity group.

**Table 2:** Perioperative outcomes and morbidity following minimally invasive antireflux surgery stratified by body mass index categories

	Body mass index—obesity class				
	Overweight 25–30 kg/m^2^ (*n* = 172)	Class I 30–35 kg/m^2^ (*n* = 125)	Class II 35–40 kg/m^2^ (*n* = 30)	Class III ≥ 40 kg/m^2^ (*n* = 11)	*p* value
Intraoperative complications					
Operative time, min	90 [70–105]	90 [75–105]	87.5 [80–113.8]	110 [72.5–150]	0.548
Estimated blood loss, mL	20 [10–25]	25 [10–30]	22.5 [15–25]	37.5 [25–93.8]*	**0.030**
Blood loss ≥ 250 mL	0 (0)	1 (0.8)	0 (0)	1 (9.1)	**0.001**
Any intraoperative complication	6 (3.5)	6 (4.8)	1 (3.3)	3 (27.3)*	**0.006**
Organ laceration/perforation	4 (2.3)	2 (1.6)	0 (0)	0 (0)	
Other complications**‡**	2 (1.2)	4 (3.2)	1 (3.3)	3 (27.3)	
ICU admission and length of stay					
ICU admission	3 (1.7)	5 (4)	1 (3.3)	2 (18.2)*	**0.026**
Hospital length of stay, days	1 [1, 2]	1 [1, 2]	1 [1, 2]	1 [1–2.5]	0.544
Early postoperative complications					
Any postoperative complication	13 (7.6)	11 (8.8)	2 (6.7)	3 (27.3)	0.153
Clavien-Dindo Grade I	5 (2.9)	4 (3.2)	1 (3.3)	0 (0)	
Clavien-Dindo Grade II	7 (4.1)	4 (3.2)	0 (0)	0 (0)	
Clavien-Dindo Grade IIIb	1 (0.6)	1 (0.8)	0 (0)	3 (27.3)	
Clavien-Dindo Grade IVa	0 (0)	2 (1.6)	1 (3.3)	0 (0)	

Bold *p* values indicate a *p* < 0.05

*HH* hiatal hernia, *ICU* intensive care unit, *ITS* intrathoracic stomach

^*^Indicates an adjusted *p* < 0.05 (via Holm-Bonferroni correction or Dunn’s test) when compared to patients with a BMI between 25 and 30 kg/m^2^

^‡^Complications including pneumothorax, vagus nerve injury, etc.

Further, despite similar LOS (i.e., hospital discharge was completed on postoperative day 1 for most patients), a higher rate (although not statistically significant) of early postoperative complications was observed among the class III obesity group (overweight: 7.6%; class I: 8.8%; class II: 6.7%; and class III: 27.3%; *p* = 0.153). Of note, all complications presented in patients with class III obesity were considered severe (Clavien–Dindo ≥ IIIa) and included 2 cases of respiratory failure requiring re-intubation and 1 case of new-onset postoperative dysphagia requiring re-operation on postoperative day 5 (i.e., take-down of Dor fundoplication). The details on the nature of other early postoperative complications are summarized in Supplementary Material–S2. No 30-day procedure-related mortalities were documented across the entire cohort.

### Midterm surgical and patient-reported outcomes

Table 3 summarizes the midterm surgical and patient-reported outcomes. Postoperative follow-up (≥ 12 months) was completed in 177 (52.4%) patients, distributed among the BMI categories: overweight (95/172, 55.2%), class I obesity (64/125, 51.2%), class II obesity (15/30, 50%), and class III obesity (3/11, 27.3%). Overall, median follow-up durations were similar across groups, ranging from 24 to 36 months. Among the entire cohort, the overall rate of HH recurrence of any size, as determined by barium esophagram or EGD, was 11/177 (6.2%). However, significant re-herniation (i.e., > 2 cm) was relatively rare and identified in only 1 (1.1%) overweight patient and 2 (2.1%) patients with class I obesity.

**Table 3:** Midterm surgical and patient-reported outcomes following minimally invasive antireflux surgery stratified by body mass index categories

	Body mass index—obesity class				
	Overweight 25–30 kg/m^2^ (*n* = 172)	Class I 30–35 kg/m^2^ (*n* = 125)	Class II 35–40 kg/m^2^ (*n* = 30)	Class III ≥ 40 kg/m^2^ (*n* = 11)	*p* value
Follow-up characteristics					
Completed f/u ≥ 12 months	95/172 (55.2)	64/125 (51.2)	15/30 (50)	3/11 (27.3)	0.324
Time to last f/u, months	29 [15–60]	25.5 [12–57]	24 [12–43]	36 [26–48]	0.631
Midterm follow-up outcomes					
Any evidence of HH recurrence†	5/95 (5.3)	6/64 (9.4)	0/15 (0)	0/3 (0)	0.702
Objective re-herniation ≤ 2 cm	4/95 (4.2)	4/64 (6.3)	0/15 (0)	0/3 (0)	
Objective re-herniation > 2 cm	1/95 (1.1)	2/64 (2.1)	0/15 (0)	0/3 (0)	
Any acid suppression therapy†	13/95 (13.7)	13/64 (20.3)	1/15 (6.7)	0/3 (0)	0.416
H2-receptors antagonist	7/95 (7.4)	3/64 (4.7)	0/15 (0)	0/3 (0)	0.640
Proton pump inhibitors	8/95 (8.4)	11/64 (17.2)	1/15 (6.7)	0/3 (0)	0.278
Patient-reported outcome measures					
Total GERD-HRQL score†	2 [0–5.8]	1 [0–10]	1.5 [0–4.8]	17 [10.5–25]	0.253
Heartburn component	0 [0–2.8]	0 [0–6]	0 [0–1.8]	8 [4–11]	0.498
Regurgitation component	0 [0–1]	0 [0–1]	0 [0]	8 [6–10.5]*	**0.036**
Dysphagia component	0 [0–1]	0 [0–2]	0 [0–1]	1 [0.5–3]	0.786
Responders to patient satisfaction	75/95 (78.9)	51/64 (79.7)	11/15 (73.3)	3/3 (100)	0.888
Patient satisfaction, scale (0–10) §	10 [8–10]	10 [9, 10]	10 [9.5–10]	7 [6–7.5]	0.076
Adequate satisfaction (score ≥ 8) **§**	61/75 (81.3)	43/51 (84.3)	10/11 (90.9)	1/3 (33.3)	0.130

Bold *p* values indicate a *p* < 0.05

*BMI* body mass index, *f/u* follow-up, *GERD-HRQL* Gastroesophageal Reflux Disease Health-Related Quality of Life, *HH* hiatal hernia

^*^Indicates an adjusted *p* < 0.05 (via Holm-Bonferroni correction or Dunn’s test) when compared to patients with a BMI between 25 and 30 kg/m^2^

^†^Reported frequencies are adjusted by the denominators of f/u completed at least 12 months after the procedure

^§^Reported frequencies are adjusted by the denominator, i.e., number of patients who responded to the elective question regarding satisfaction with the surgical procedure

The use of any form of acid suppression therapy decreased after ARS across all groups and did not differ significantly between groups at last available follow-up (overweight: 13.7%; class I: 20.3%; class II: 6.7%; and class III: 0%; *p* = 0.416). The specific distributions of H2-receptor antagonist and PPI use was similarly distributed. Further, despite sustained improvements in quality of life in most patients, a slightly worse total median GERD-HRQL score was observed within the class III group (overweight: 2; class I: 1; class II: 1.5; class III: 17; *p* = 0.253); this result was driven mainly due to a higher regurgitation subcomponent score (Table 3).

Likewise, overall midterm patient satisfaction scores were notably high, with median values ranging from 7 (class III obesity) to 10 (rest of the groups). Moreover, 115 of 149 (82.1%) patients who responded to this elective question reported adequate satisfaction (i.e., ≥ 8 on the 0–10 scale). Of note, while no statistically significant differences were observed across BMI groups, a relatively lower rate of adequate satisfaction was observed in the class III group (overweight: 81.3%; class I: 84.3%; class II: 90.9%; class III: 33.3%; *p* = 0.130).

## Discussion

The rising prevalence of obesity is causing an increased number of referrals of overweight and obese patients with GERD to specialized foregut surgery clinics. While bariatric surgery at the time of HH repair is the preferred approach as it controls reflux and provides metabolic benefits, some individuals undergo laparoscopic fundoplication given ineligibility for bariatric programs, insurance coverage, or patient preference. Consequently, we analyzed perioperative and midterm ARS outcomes in a large cohort of overweight and obese patients and observed several key findings: (i) during the study period, up to 32.6% of patients undergoing elective primary ARS were overweight and up to 31.4% were obese; (ii) minimally invasive ARS demonstrated an adequate safety profile, low midterm recurrence rates, and quality of life improvements across overweight and class I obese populations; (iii) patients with class II obesity (BMI 35–40 kg/m^2^) exhibited fairly similar outcomes to those within lower BMI categories; and (iv) patients with class III obesity (BMI ≥ 40 kg/m^2^) experienced slightly increased morbidity and worse patient-reported outcomes.

Historically, it has been argued that as a higher BMI is associated with more numerous and severe comorbidities, surgical risk—regardless of the type of procedure—will be greater. Nevertheless, with the introduction of laparoscopy, this proposition was subject to change, as the perceived advantages of minimally invasive gastrointestinal surgery (particularly its role in mitigating postoperative morbidity) began to become more obvious within cohorts of patients with obesity [19]. However, initial experiences as well as clinical studies from the early laparoscopic era led to controversial and often polemic findings [15], which we believe are still a major contributor to surgical hesitancy in offering ARS as definitive treatment for GERD to overweight and obese patients.

Among these early observational studies, Perez et al. [15] observed significantly higher reflux recurrence (8/35, 22.9%) in the obese group compared to normal-weight (4/78, 5.1%) and overweight (4/74, 5.4%) groups among 187 patients who underwent laparoscopic Nissen fundoplication. Conversely, Campos et al. [20] documented similar rates of symptomatic recurrence after laparoscopic Nissen fundoplication in normal-weight patients (6/47, 12.8%) and those with BMI > 25 kg/m^2^ (20/152, 13.6%) over a median follow-up of 15 months. Similarly, Fraser et al. [21] examined 194 patients undergoing the same procedure and found no association between obesity and adverse operative outcomes; in fact, the authors reported a trend of diminished satisfaction among normal-weight patients.

In terms of postoperative morbidity and procedure safety, our findings are consistent with more recent literature. We observed more blood loss and higher rates of intraoperative complications and ICU admissions only in patients with morbid obesity (BMI > 40 kg/m^2^), whereas morbidity remained comparable across all patients with BMI 25–40 kg/m^2^. This aligns with the retrospective review by Telem et al. [22], which analyzed 4231 laparoscopic fundoplication patients and found that those with BMI ≥ 35 kg/m^2^ experienced longer operative times (129.7 vs. 118 min) but no significant differences in overall complication rates (2 vs. 1.9%) or length of stay (1.8 vs. 1.8 days) compared to patients with BMI < 35 kg/m^2^. Similarly, Luketina et al. [23] retrospectively compared patients with normal BMI (20–25 kg/m^2^, *n* = 34) and obesity (BMI ≥ 30 kg/m^2^, *n* = 36) and concluded that obesity was not associated with worse clinical or objective outcomes after laparoscopic antireflux surgery. Moreover, a propensity score-matched cohort study by Hoshino et al. [24], which included 105 patients each in BMI < 25 and BMI ≥ 25 kg/m^2^ groups, also reported slightly longer operative times in the overweight-obese group but no difference in intraoperative complications.

Interestingly, we observed relatively low objective recurrence rates within the first postoperative year (5.3–9.4% for any recurrence; 1.1–2.1% for recurrence of HH > 2 cm) alongside significant and sustained quality-of-life improvements in most overweight and obese patients. This contrasts with the 2019 meta-analysis by Bashir et al. [25], which analyzed 3346 fundoplication patients from 12 studies (2423 patients with BMI < 30 kg/m^2^ vs. 923 patients with BMI > 30 kg/m^2^) and reported a recurrence risk ratio of 1.36 (95% CI 1.08–1.72, *p* = 0.009) for obese patients. However, careful interpretation of this aggregate risk is necessary. First, there was substantial heterogeneity in follow-up periods (6–77 months) and surgical techniques among included studies. Second, and perhaps more importantly, after detailed review of the only 5 studies that reported objective recurrence, only the study from Perez et al.[15] (in the early laparoscopic era) reported a recurrence rate > 20% among obese patients. In contrast, in the other 4 studies, recurrence ranged from 1.4 to 10%, which was comparable to the 0–10.4% observed among non-obese individuals [26–29]. This closer look suggests a major temporal bias, implying that contemporary fundoplication techniques in patients with BMI > 30 kg/m^2^ might yield recurrence rates comparable to those observed in non-obese populations.

Our study did not aim to establish equivalence to bariatric surgery, to prove that minimally invasive fundoplication might have comparable outcomes to bariatric surgery, or to diminish the advantages conferred by bariatric procedures [30, 31]. Rather, our goal was to assess whether durable management of GERD could be achieved in a population facing a theoretical increased risk of procedural failure (i.e., due to higher intra-abdominal pressure) when bariatric surgery is not an option.

Our study carries clinical implications. Our results suggest that, particularly in patients with class II obesity (BMI 35–40 kg/m^2^), where bariatric procedures are typically indicated, the trade-off between surgical risk and procedural benefits in terms of reflux control and related quality of life appears promising and durable, indeed similar to that achieved in patients with BMI < 30 kg/m^2^ and those with class I obesity. Despite our sample size limitations within class II obesity patients, our study provides exploratory observational data indicating that minimally invasive ARS should be at least considered among these individuals if bariatric surgery is not an option due to insurance issues or patient preferences. This approach, however, must be complemented by other clinical considerations, including comprehensive weight management strategies and lifestyle modification counseling. By the same reasoning, our study highlights potentially increased risks in patients with morbid obesity (BMI ≥ 40 kg/m^2^); hence, straightforward ARS in this subgroup should be carefully considered only if preoperative weight management strategies have failed and after thorough patient education and shared decision-making regarding realistic outcomes and risks. Finally, we believe that only experienced foregut surgeons should offer such counseling as well as perform the procedure.

Our study has certain limitations. First, its retrospective design may introduce selection bias, as the precise reasons for not proceeding with bariatric surgery in class II–III patients were not retrieved; however, based on our experience, most cases are likely due to lack of insurance coverage of bariatric procedures or patient’s unwillingness to take the perceived “drastic” step of bariatric surgery. Second, this study is limited by incomplete midterm follow-up and small subgroup sample sizes. At ≥ 12 months, follow-up data were available for only 52.4% of the overall cohort, and despite routinely ordering postoperative barium swallow studies, imaging was available in only 45.8% of patients, thereby reducing statistical power, particularly for comparisons of hiatal hernia recurrence. These limitations are compounded by the absence of adjusted analyses considering potential confounders (e.g., age, sex, and preoperative comorbidity burden) in a clinically heterogeneous cohort that included different fundoplication techniques. This is especially important in the class II–III obesity groups, in whom the subgroup size was very small, and follow-up was particularly limited, increasing the risk of attrition bias and restricting the strength and generalizability of conclusions in this population. Third, it could be argued that a more ideal comparator group would be patients with a “normal” BMI (20–25 kg/m^2^). However, ARS is routinely performed and considered standard surgical treatment for GERD in overweight patients (25–30 kg/m^2^), and we showed that the outcomes for class I and II obesity groups were similar to those of the overweight group. Fourth, the generalizability of our results might be somewhat limited, as the outcomes presented are derived from procedures performed by an experienced foregut surgeon in a high-volume institution. Finally, we did not investigate weight changes over time or changes in specific therapies directed to weight loss/control; therefore, we are unable to determine if minimally invasive ARS had a secondary metabolic impact or if confounding weight fluctuations influenced long-term reflux outcomes.

## Conclusion

Primary elective minimally invasive fundoplication offers a durable and effective therapeutic option for GERD in patients with BMI 25–40 kg/m^2^. This approach appears to provide a favorable safety profile, effective reflux control, and improved quality of life in the midterm, achieving similar outcomes between patients in the overweight, class I obesity, and class II obesity groups. Importantly, patients with class II obesity (i.e., 35–40 kg/m^2^) might benefit from straightforward ARS without increased procedural risk, particularly when bariatric surgery is not feasible.

## Supplementary Information

Below is the link to the electronic supplementary material.

Supplementary file1 (DOCX 25 KB)

## Data Availability

The data analyzed in this study are stored in an institutional REDCap database. Access to this dataset is subject to IRB review/approval requirements; all inquiries should be directed to the corresponding author.

## Abbreviations

AOH: Angle of His
ARS: Antireflux surgery
BMI: Body mass index
EGD: Esophagogastroduodenoscopy
EndoFLIP: Endoscopic functional luminal impedance planimetry
GERD: Gastroesophageal reflux disease
GERD-HRQL: Gastroesophageal Reflux Disease Health-Related Quality of Life
HH: Hiatal hernia
ICU: Intensive care unit
IQR: Interquartile range
LOS: Length of stay
PPI: Proton pump inhibitor
RYGB: Roux-en-Y gastric bypass
